# Intact Cytoskeleton Is Required for Small G Protein Dependent Activation of the Epithelial Na^+^ Channel

**DOI:** 10.1371/journal.pone.0008827

**Published:** 2010-01-21

**Authors:** Alexey V. Karpushev, Daria V. Ilatovskaya, Tengis S. Pavlov, Yuri A. Negulyaev, Alexander Staruschenko

**Affiliations:** 1 Department of Physiology, Medical College of Wisconsin, Milwaukee, Wisconsin, United States of America; 2 Kidney Disease Center, Medical College of Wisconsin, Milwaukee, Wisconsin, United States of America; 3 Institute of Cytology, Russian Academy of Sciences, St. Petersburg, Russian Federation; Dresden University of Technology, Germany

## Abstract

**Background:**

The Epithelial Na^+^ Channel (ENaC) plays a central role in control of epithelial surface hydration and vascular volume. Similar to other ion channels, ENaC activity is regulated, in part, by cortical cytoskeleton. Besides, the cytoskeleton is an established target for small G proteins signaling. Here we studied whether ENaC activity is modulated by changes in the state of the cytoskeleton and whether cytoskeletal elements are involved in small G protein mediated increase of ENaC activity.

**Methods and Findings:**

First, the functional importance of the cytoskeleton was established with whole-cell patch clamp experiments recording ENaC reconstituted in CHO cells. Pretreatment with Cytochalasin D (CytD; 10 µg/ml; 1–2 h) or colchicine (500 µM; 1–3 h) to disassembly F-actin and destroy microtubules, respectively, significantly decreased amiloride sensitive current. However, acute application of CytD induced rapid increase in macroscopic current. Single channel measurements under cell-attached conditions revealed similar observations. CytD rapidly increased ENaC activity in freshly isolated rat collecting duct, polarized epithelial mouse mpkCCD_c14_ cells and HEK293 cells transiently transfected with ENaC subunits. In contrast, colchicine did not have an acute effect on ENaC activity. Small G proteins RhoA, Rac1 and Rab11a markedly increase ENaC activity. 1–2 h treatment with colchicine or CytD abolished effects of these GTPases. Interestingly, when cells were coexpressed with ENaC and RhoA, short-term treatment with CytD decreased ENaC activity.

**Conclusions:**

We conclude that cytoskeleton is involved in regulation of ENaC and is necessary for small G protein mediated increase of ENaC activity.

## Introduction

Regulation of sodium reabsorption is one of the most important questions in the area of kidney physiology. The rate limiting step for Na^+^-(re)absorption across many epithelia, including those in the distal nephron, lungs and colon is the activity of ENaC that plays a central role in salt homeostasis support and blood pressure control [1], [2]. Transport processes based on ENaC functioning play a pivotal role in homeostasis of epithelial kidney tissues and other organs. Abnormalities in ENaC function have been linked to disorders of total body Na^+^ homeostasis, blood volume, blood pressure, and lung fluid balance [3], [4].

Cytoskeletal elements are evidently an important part of ion transport regulation in epithelia, but the functional role of actin filaments in membrane transport protein-cytoskeleton interactions is not clear yet. The cytoskeleton is a dynamic structure which plays an essential role in regulation of cellular events including the stability of cell shape, the distribution of integral membrane proteins and the control of hormone action. It has been shown that actin microfilaments are implicated in diverse cell functions including endo- and exocytosis, membrane trafficking and cell migration, distribution of integral membrane proteins, regulation of cells morphology and even mechanical gating during osmosensory transduction [5]. It was previously shown that actin cytoskeleton and microtubules play an important role in the regulation of membrane transport processes in epithelia [6]–[11].

Biophysical data suggesting an interaction of ENaC with actin were first presented by Cantiello et al [10]. Using patch clamp analysis, they observed an increase in amiloride-sensitive sodium channel activity following disruption of actin-based cytoskeleton in *Xenopus laevis* A6 cells [10]. Later it was shown that ENaC can directly interact with actin [9]. It has been shown that F-actin and ENaC colocalize at both the apical membrane and within the subapical cytoplasm [12]. Colocalization of α-ENaC and F-actin in the subapical cytoplasm suggests that in addition to regulating ENaC activity, a direct interaction between F-actin and ENaC may function in intracellular trafficking of ENaC from a subapical pool to the plasma membrane in response to stimulation by hormones, such as vasopressin and insulin [9]. Likewise, BK channel activation by insulin is blocked by actin filament stabilization [13].

The site and the duration of actin polymerization are tightly controlled by small G proteins [14]. Recent findings indicate that activity of ion channels can also be controlled by small G proteins [15]. Signaling processes mediated by small G proteins can impinge upon the activity of a wide variety of membrane-resident ion channels. In some cases, small GTPases interact directly with ion channels to elicit regulation, and, in others, regulation is mediated by intermediary signaling proteins. We and others have shown that small GTPases including K-Ras, RhoA, Rac1 and several Rabs are involved in regulation of both gating and the number of ENaCs in the apical plasma membrane [16]–[22].

The modulatory role of actin filaments in ENaC functioning has been described in A6 cells [10], *Xenopus* oocytes and planar lipid bilayers [23], [24]. The role of the cytoskeleton in the regulation of ENaC in native tissues is unclear. While there are many studies demonstrating cytoskeletal filament regulation of ENaC in heterologous systems, the interactions of ENaC with cytoskeleton in native epithelial cells have not been directly explored and are one of the focuses of this study. Cytoskeleton remodeling provides the forces required for a variety of cellular processes based on membrane dynamics, such as endocytosis, exocytosis, and vesicular trafficking at the Golgi. All these events are coordinated by networks of associated proteins. Furthermore, the role of the cytoskeleton in the modulation of ENaC by small G proteins has never been investigated. In the present study we report that tubulin and actin are involved into regulation of ENaC in principal cells and are necessary for small G protein-mediated activation of ENaC.

## Results

### Biphasic Effect of Cytochalasin D on ENaC Activity

**Figure 1:**
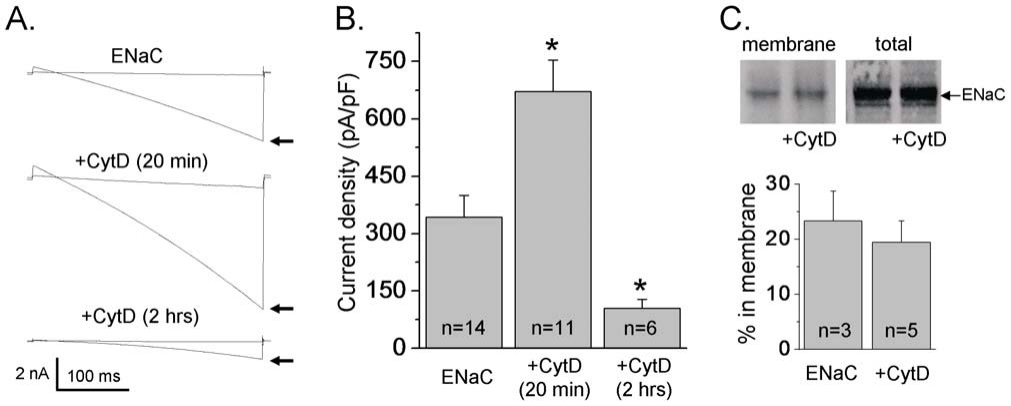
Biphasic effects of Cytochalasin D on ENaC activity. A, Overlays of typical macroscopic current traces before (arrow) and after 10 µM amiloride from voltage-clamped CHO cells transfected with α, β and γ subunits of mENaC. Currents evoked with a voltage ramp (60 to −100 mV from a holding potential of 40 mV). Inward Na^+^ currents are downward. Whole-cell current traces without treatment with Cytochalasin D (CytD, 10 µg/ml) (top) and after 20 min (middle) and 2 hrs (bottom) of treatment with CytD are shown. B, Summary graph of amiloride-sensitive current density at −80 mV for CHO cells expressing α, β and γ subunits of mENaC without treatment with CytD, after 20 min and 2 hrs of treatment with CytD, respectively. The number of observations for each group is shown. *, *versus* no treatment. C, Typical Western blot probed with anti-Myc antibody containing the plasma membrane fraction and total cell lysate from CHO cells expressing Myc-tagged α, β, and γENaC in the absence and presence of treatment with CytD (10–20 min). Plasma membrane proteins were labeled with sulfo-NHS-LC-biotin and isolated with streptavidin precipitation. Summary graph showing percent ENaC in the plasma membrane in cells expressing ENaC in the absence and presence of treatment with CytD is also shown. Percent ENaC in the membrane was established with densitometry of Western blots with the number of independent experiments indicated. For each experiment, the density of the membrane fraction of ENaC was divided by the total cellular pool of ENaC.

Initial patch clamp experiments were employed to determine the influence of actin filament disruption with cytochalasin D (CytD, 10 µg/ml) on ENaC activity in Chinese hamster ovary (CHO) cells transiently transfected with α-, β- and γ-subunits of mouse ENaC (mENaC). Figure 1A shows typical macroscopic current traces from whole cell experiments before (arrow) and after treatment with amiloride (10 µM) in control CHO cells without treatment (top) and CHO cells treated with CytD for 20 min (middle) and 2 hrs (bottom), respectively. Currents were elicited by voltage ramping from 60 mV down to −100 mV (holding potential 40 mV). As summarized in Figure 1B, short term exposure to CytD markedly increased ENaC activity, whereas long term treatment showed its consistent decrease compared to control experiments. Average amiloride-sensitive current density in control experiments (without treatment with CytD) was 342±57****pA/pF (n = 14). Disruption of actin microfilaments for 20 min with CytD significantly increased current density to 671±82****pA/pF (n = 11). In contrast, 2 hrs treatment with CytD resulted in significant decrease of ENaC activity to 105±24 pA/pF (n = 6) (Figure 1B). We interpret these results as showing that acute disrupting of actin cytoskeleton enhances ENaC activity but longer exposures to CytD result in changes in cell shape and loss of plasma membrane interactions with the cytoskeleton and, correspondingly, decrease of ENaC activity. Similarly, Cantiello et al., have shown in A6 cells that Na^+^ channels activation by CytD was present only in cells exposed to CytD for <40 min. Cells exposed to CytD for short periods of time were devoid of macroscopic changes in cell shape. However, longer exposures to the drug resulted in changes in cell shape and loss of plasma membrane interactions with actin [10], [25].

Figure 1C shows a representative Western blot probed with anti-Myc antibody containing whole cell lysate from cells overexpressing Myc-tagged ENaC not treated and treated for 10–20 min with CytD. The membrane fractions and total cellular pools of ENaC are shown for each group. The membrane fraction was isolated by streptavidin precipitation of total cell lysates created from cells that had their plasma membrane proteins labeled with sulfo-NHS-LC-biotin. This approach reliably separates integral plasma membrane from cytosolic proteins. As summarized, the percentage of ENaC in the plasma membrane in cells expressing the channel alone was 23.3±5.4%. Acute treatment with CytD had no effect on the membrane levels of ENaC (19.4±3.9%). Thus, as shown in Figure 1C, membrane ENaC levels are not influenced by acute treatment with CytD and these data are most consistent with acute destroying of the actin cytoskeleton impinging upon ENaC open probability.

### Acute Disruption of Actin Microfilaments with CytD Markedly Increases ENaC Activity (*NP_o_*) in HEK293 Cells

**Figure 2:**
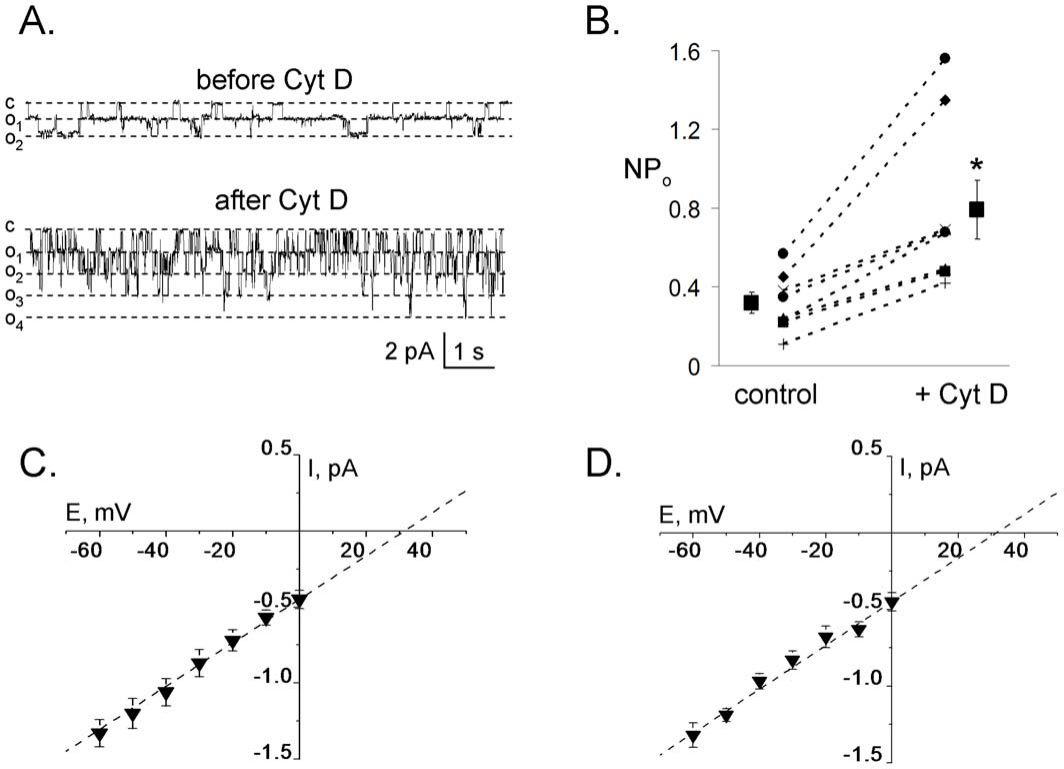
Acute application of Cytochalasin D causes an increase in ENaC activity. A, Representative current traces from a cell-attached patch in a HEK293 cell transfected with α-, β- and γ-mENaC subunits before and after addition of Cytochalasin D (CytD, 10 µg/ml) to the bath solution. This patch was held at a −60 mV test potential during the course of the experiment. Dashed lines indicate the respective current levels shown to the left. “c” and “o” denote corresponding closed and open current levels. B, Summary graph of ENaC channel activity (*NP_o_*) changes in response to CytD from paired cell-attached experiments performed on HEK293 cells transiently transfected with all three ENaC subunits. *, *versus* before application of CytD. C and D, Single-channel current-voltage relation for ENaC in cell-attached patches made in HEK293 cells transfected with α-, β- and γ-mENaC subunits before (C) and after (D) 10 min treatment with CytD. Points in the plots are mean ± SEM for at least nine experiments at each potential.

To further test the effect of CytD on ENaC activity at a single channel level we attempted patch clamp experiments in cell-attached configuration on HEK293 cells transiently transfected with α-, β- and γ-mENaC. Figure 2A demonstrates current traces from a cell-attached patch from a HEK293 cell expressing ENaC before and after application of CytD (10 µg/ml) at a holding potential –60 mV. The addition of CytD quickly increased ENaC activity. As it is clearly seen from these representative current traces and summary graphs (Figure 2B), destroying of actin filaments with CytD significantly increased the *NP*_o_ of ENaC within several minutes. As summarized in Figure 2B, *NP*_o_ has increased 2.7 fold after 10 min treatment with CytD. We next tested whether CytD influences single channel currents and conductance of ENaC. Figures 2C and D show the single channel current–voltage relation for HEK293 cells transiently transfected with mENaC subunits before and after treatment with CytD (10 µg/ml; 10 min). The mean current-voltage relationship of single ENaC was not affected by application of CytD. Neither unitary current conductance (14.7±0.5 and 14.8±0.4 pS before and after CytD, respectively) nor reversal potential (35.2±0.1 and 35.9±0.2 mV before and after addition of CytD, respectively) were not altered by disrupting of actin filaments.

### Acute Disruption of Actin Microfilaments with CytD Enhances ENaC *NP_o_* in Native Principal Cells

**Figure 3:**
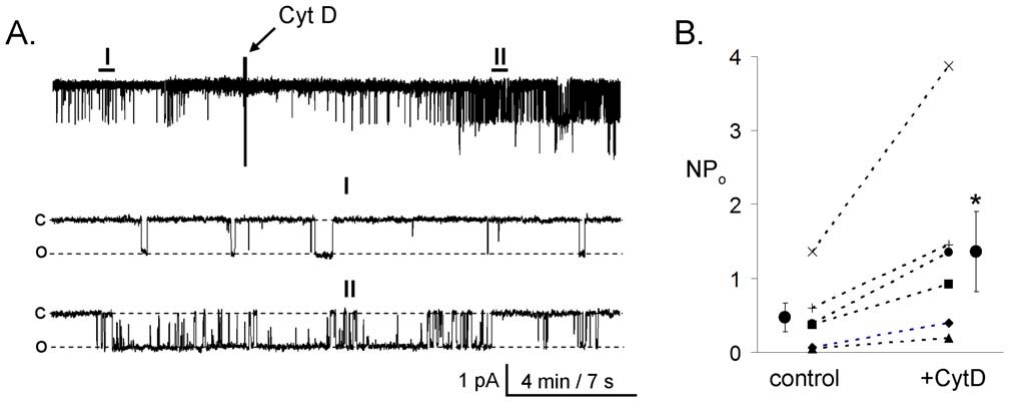
Cytochalasin D rapidly increases ENaC activity in the apical membrane of mpkCCD_c14_ principal cells. A, Continuous current trace from a representative cell-attached patch that was made on the apical membrane of mpkCCD_c14_ principal cells before and after treatment with CytD. Areas before (I) and after (II) treatment are shown below with an expanded time scale. This patch was held at a −60 mV test potential during the course of the experiment. “c” and “o” denote closed and open current level, respectively. B, Summary graph of *NP_o_* in cell-attached patches in mpkCCD_c14_ cells before (control) and after (+CytD) treatment with CytD. *, *versus* before application of CytD.

Since the previous and published results supported coupling between ENaC activity and actin filaments, we were interested in testing whether such a mechanism plays a role in physiologic regulation of the channel in native and cultured principal cells. First, we performed similar experiments with immortalized mouse cortical collecting duct principal cells (mpkCCD_c14_). Polarized epithelial monolayers of mpkCCD_c14_ cells were grown on permeable support and produced robust transepithelial ion transport. ENaC activity was continuously monitored in paired cell-attached experiments. Extracellular application of CytD (10 µg/ml) rapidly increased ENaC *NP*_o_ within a couple of minutes. A representative current trace from a cell attached patch showing such activation is reported in Figure 3A. An arrow denotes addition of the reagent to the bath solution. A continuous trace before and after addition of CytD is shown at the top. Segments before (I) and after CytD (II) are shown below at expanded time scales. As summarized in Figure 3B, CytD acutely increases ENaC *NP*_o_ within 10 min, from 0.47±0.20 to 1.37±0.54 (*n* = 6).

**Figure 4:**
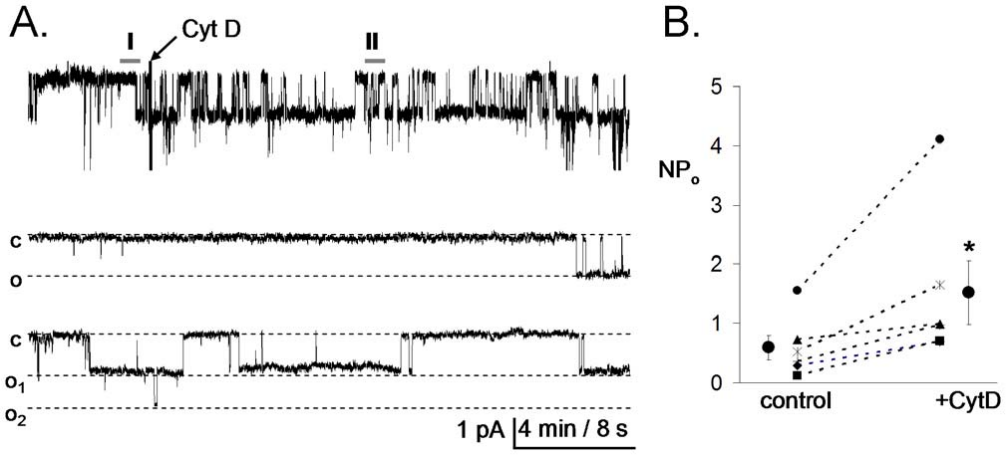
Cytochalasin D rapidly increases ENaC activity in principal cells in isolated split-open rat collecting ducts. A, Continuous current trace from a representative cell-attached patch that was made on the apical membrane of principal cells in isolated split-open rat collecting ducts before and after treatment with cytochalasin D (CytD). Areas before (I) and after (II) treatment are shown below with an expanded time scale. This patch was held at a −60 mV test potential during the course of the experiment. “c” and “o_i_” denote closed and open current levels, respectively. B, Summary graph of *NP_o_* in cell-attached patches from freshly isolated CCD cells before (control) and after acute addition of CytD. *, *versus* before application of CytD.

The representative current recordings in Figure 4A document the effect of disrupting of actin filaments on ENaC *NP*_o_ in freshly isolated rat collecting duct principal cells. The representative patch in Figure 4A (one of six) formed on the apical membrane of a native principal cell was clamped with a −60 mV test potential and contained a single ENaC. Again, a continuous trace before and after addition of CytD is shown at the top. Segments before and after CytD are shown below at expanded time scales. As is apparent in this representative trace and summarized in Figure 4B, application of CytD resulted in a rapid increase in ENaC *NP_o_* in this native preparation. Thus, the results in Figures 3 and 4 demonstrate that the *NP*_o_ of active ENaC within the apical membrane of native and cultured principal cells is tightly coupled to the actin cytoskeleton.

### Effect of Colchicine on ENaC Activity

**Figure 5:**
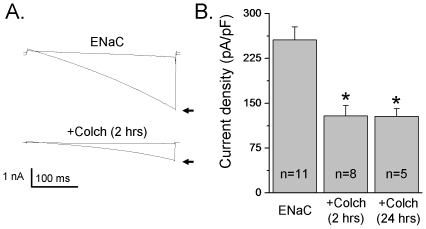
Destroying of microtubule network with colchicine decreases ENaC activity. A, Overlays of typical macroscopic current traces before (arrow) and after 10 µM amiloride from voltage-clamped CHO cells transfected with α-, β- and γ-mENaC subunits without (top) and with treatment (bottom) with colchicine (Colch, 500 µM). B, Summary graph of amiloride-sensitive current density at −80 mV for CHO cells expressing mENaC without treatment with colchicine, after 2 and 24 hrs of treatment with colchicine, respectively. The number of observations for each group is shown. *, *versus* untreated cells.

It is well established that microtubules and their associated proteins are important determinants of regulation of ion channels. Thus, we next tested whether disruption of microtubules influences ENaC macroscopic currents in CHO cells transiently transfected with mENaC. First we treated cells for 10–20 min with colchicine to examine acute effect on ENaC activity. Colchicine inhibits microtubule polymerization by binding to tubulin, one of the main constituents of microtubules. Short term treatment with colchicine (500 µM) did not affect neither macroscopic nor single channel ENaC activity (data not shown). However, long term treatment with colchicine significantly decreased amiloride sensitive current density in CHO cells transiently transfected with mENaC. Cells were treated with colchicine (500 µM) for 2 or 24 hrs. Figure 5A shows macroscopic ENaC currents before and after treatment with amiloride in CHO cells expressing mENaC not treated (top) and treated for 2 hrs with colchicine (bottom). As summarized in Figure 5B, pretreatment with the microtubules inhibitor colchicine significantly decreased ENaC activity from 256±22 to 129±17 and 128±13 pA/pF (after 2 and 24 hrs treatment, respectively). Together, these results show that ENaC activity is controlled by changes in microtubules network.

### Intact Cytoskeleton Is Required for Small G Protein Dependent Activation of ENaC

**Figure 6:**
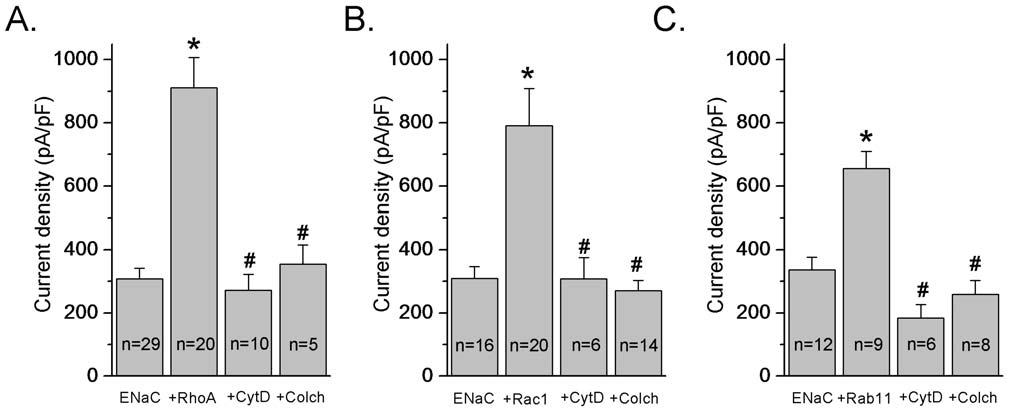
Long term treatment with Cytochalasin D and colchicine abolished small G protein-dependent increases in ENaC activity. A–C, Summary graphs of amiloride-sensitive current density at −80 mV for CHO cells expressing either mENaC alone or coexpressed with small GTPases RhoA (A), Rac1 (B) or Rab11 (C) in the absence and presence of treatment with CytD (10 µg/ml; 2 hrs) or colchicine (500 µM; 2 hrs). The number of observations for each group is shown. *, *versus* ENaC alone. ^#^, *versus* corresponding small G protein.

Our previous studies showed that several small G proteins, including RhoA, Rac1 and Rab11a alter ENaC activity [17], [18], [20], [26]. Small G proteins are believed to be key regulators of the cytoskeleton. Thus, we were interested in testing the hypothesis that cytoskeleton is involved in small GTPases mediated changes of ENaC activity. First, to investigate the long term actions of agents disrupting actin filaments or microtubules we reconstituted the channel in CHO cells in the absence and presence of co-expressed constitutively active RhoA (G14V), Rac1 (QL) or wild type Rab11a. As shown in Figure 6, cotransfection of ENaC with RhoA, Rac1 and Rab11a leads to a significant increase in ENaC activity. Current densities for mENaC alone were 308±33 (n = 29), 309±37 (n = 16) and 336±40 (n = 12) pA/pF for control groups (Figures 6 A, B and C, respectively). Coexpression of small G proteins resulted in significant increases of ENaC activity to 912±96, 791±117 and 655±54 pA/pF for ENaC plus RhoA, Rac1 and Rab11a, respectively. Transfected cells coexpressing mENaC and small G proteins were then treated with CytD (10 µg/ml) or colchicine (500 µM) for 2 hrs. Figure 6 summarizes decreases in ENaC activity under treatment with both reagents in CHO cells coexpressed with RhoA, Rac1 and Rab11a. As clearly seen in Figure 6, effects of RhoA, Rac1 and Rab11a were abolished by disruption of actin or microtubular networks.

**Figure 7:**
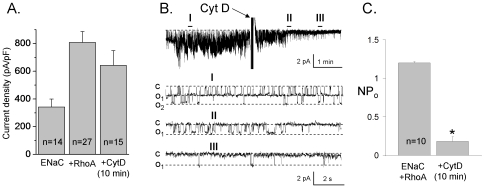
Cytochalasin D decreases ENaC activity when the channel coexpressed with RhoA. A, Summary graph of macroscopic amiloride-sensitive current density for CHO cells expressing either mENaC alone (ENaC) or coexpressing constitutively active RhoA^G14V^ (+RhoA) before and after treatment with CytD (10 µg/ml; 10 min). The number of observations for each group is shown. B, Continuous current trace from a representative cell-attached patch in HEK293 cells coexpressing α-, β- and γ- mENaC subunits and RhoA^G14V^ before and after treatment with CytD. Areas before (I) and after (II, III) treatment are shown below with an expanded time scale. This patch was held at a −60 mV test potential during the course of the experiment. “c” and “o_i_” denote closed and open current levels, respectively. C, Summary graph of *NP_o_* in cell-attached patches from HEK293 cells coexpressing mENaC and RhoA^G14V^ before and after treatment with CytD for 10 min. *, *versus* untreated cells.

Surprisingly, short term application of CytD to cells expressing both mENaC and constitutively active RhoA^G14V^ did not increase macroscopic current density compared to cells that were treated with vehicle. As demonstrated in Figure 7A, treatment of CHO cells coexpressed with ENaC and RhoA^G14V^ for 10 min with CytD resulted in small but not significant decrease of ENaC activity. Current density was 343±57, 808±79 and 643±107 for ENaC alone and ENaC plus RhoA not treated and treated with CytD for 10 min, respectively (Figure 7A).

Furthermore, single channel analysis revealed that CytD decreases the *NP*_o_ when ENaC is coexpressed with RhoA. Figures 7B and C show representative current traces and summary graphs of *NP*_o_ in paired experiments for ENaC in HEK293 cells transfected with α-, β-, and γ-mENaC subunits and RhoA^G14V^ before and after treating with CytD. The representative current recording in Figure 7B documents the time course of inhibiting of ENaC activity. A continuous trace before and after addition of 10 µg/ml of CytD is shown at the top. Segments before (I) and after CytD (II and III) are shown below at expanded time scales. As is apparent from this representative trace, CytD application rapidly decreases the activity of ENaC. As summarized in Figure 7C, CytD decreased ENaC *NP_o_* from 1.20±0.01 to 0.18±0.06 (n = 10). These results are consistent with ENaC activity being acutely decreased by CytD when coexpressed with RhoA.

## Discussion

Activity of ENaC is controlled by different regulatory mechanisms and signaling molecules. Moreover, it has been shown that ENaC activity is partly regulated by cytoskeleton that is an established target for small G proteins signaling. The present study has been designed to investigate the relation between small GTPases-dependent activity of ENaC and cytockeletal state of the cell. While interaction of cytoskeleton with ENaC may serve as a means to control the distribution of ion channels, the possibility also exist that cytoskeleton and small GTPases dependent ENaC regulation pathways intersect.

Members of the Rho family of GTPases, RhoA, Rac1, and Cdc42 have been shown to influence the cytoskeletal organization in a variety of cell types. With regard to renal pathology, there is increasing evidence for a role of RhoA-Rho associated kinase signaling [27], [28]. We have shown [17], [18], [20] that morphological alterations and activation of RhoA strongly affect ENaC activity by promoting trafficking of the channel to the plasma membrane. Rac1 and Rab11a most likely also increase the number of channels at the plasma membrane [20], [26].

Moreover, Bruewer et al. have shown that Rho family GTPases regulate epithelial intercellular junctions in MDCK cells via distinct morphological and biochemical mechanisms and that perturbations in barrier function reflect any imbalance in active/resting GTPase levels rather than simply loss or gain of GTPase activity [29]. RhoA, Rac1, and Cdc42 GTPases induced time-dependent disruptions in epithelial gate function and distinct morphological alterations in apical and basal F-actin pools.

Several studies have presumed that ENaC activity and conductance are regulated through a direct interaction with actin cytoskeleton [9], [12], [23], [24], [30]. Our single channel measurements in HEK293, mpkCCD_c14_ cells and in freshly isolated CCDs have confirmed that acute effect of CytD is mediated by changes in channel activity (*NP*_o_). Furthermore, disrupting of actin cytoskeleton did not affect channel conductance. However, the colocalization of α-ENaC and F-actin in the subapical cytoplasm of MDCK cells stably expressing ENaC subunits suggest that in addition to regulating ENaC activity, a direct interaction between F-actin and ENaC may function in the intracellular trafficking of ENaC from a subapical pool to the plasma membrane [9], [12]. Moreover, Els and Chou have shown that disruption of the microfilaments with cytochalasin B resulted in a marked reduction in the recruitment of channels from a cytoplasmic pool [31]. Similarly, Butterworth et al. recently proposed that an intact actin cytoskeleton is required for ENaC insertion into plasma membrane, but is not required for subsequent channel endocytosis [32].

Our patch-clamp experiments at a whole-cell level demonstrated that 1–2 hrs treatment with colchicine or CytD abolished effects of RhoA, Rac1 and Rab11a. With regard to this finding we can deduce that effects of small G proteins on ENaC are mediated by actin and tubulin state of the cell; it is likely that pathways that lead to activation of ENaC by Rab11a, RhoA and Rac1 by affecting the number of channels at the plasma membrane involves the cytoskeleton as an essential stage of signal transduction.

Surprisingly, in cells coexpressing ENaC and RhoA no acute increase in ENaC activity after disruption of F-actin measured either under whole-cell or cell-attached conditions were observed. Stockand et al. recently reported that RhoA, likely through effects on the cytoskeleton, promotes ENaC trafficking to the plasma membrane to increase channel membrane levels and activity [18]. Our data provides further support for this position and introduces a new line of thought regarding ENaC trafficking towards the cell membrane. Consequently, our results provide evidence for the hypothesis that ENaC regulation by small G proteins is related to cytoskeletal state of the cell. Thus, changes in cytoskeleton organization may lend themselves responsible for ENaC regulation; state of actin and tubulin filament networks could be at the center of a regulatory mechanism coupled with small G protein signaling pathway, although the exact functional role of small GTPases in this mechanism requires further investigation. To summarize, we conclude that actin filaments and tubulin are involved into regulation of ENaC and are necessary for small G protein-mediated activation of ENaC.

## Materials and Methods

### Ethics Statement

Animal use and welfare adhered to the NIH *Guide for the Care and Use of Laboratory Animals* following a protocol reviewed and approved by the IACUC.

### cDNA Constructs and Cell Culture

All chemicals and materials were purchased from Fisher Scientific, Sigma or CalBiochem unless noted otherwise. CHO cells were obtained from ATCC and maintained with standard culture conditions (DMEM, 10s% FBS, 1× Penicillin-Streptomycin, 37°C, 5% CO_2_). HEK293 cells were obtained from Cell Culture Collection, Institute of Cytology, St. Petersburg, Russia and cultured in DMEM supplemented with 10% FBS, 80 µg/ml gentamicin, and 2 mM glutamine in standard conditions (37°C, 5% CO_2_). Immortalized mouse cortical collecting duct (mpkCCD_c14_) principal cells were kindly provided by Dr. A. Vandewalle, (INSERM, Paris, France) and grown on permeable supports (Costar Transwell; 0.4 µm pore, 24 mm diameter) as described previously [33], [34]. Growth medium was composed of equal volumes DMEM and Ham's F_12_, 60 nM Na^+^ selenate, 5 µg/ml transferrin, 50 nM dexamethasone, 1 nM triiodothyronine, 10 ng/ml EGF, 5 µg/ml insulin, 2% FBS and 100 µg/ml Penicillin-Streptomycin. The expression vectors encoding RhoA^G14V^ and Rab11a were from the UMR cDNA Resource Center (http://www.cdna.org). Expression vector encoding constitutively active Rac1 QL was from Dr. A. Chan (Medical College of Wisconsin). Mammalian expression vectors encoding α-, β- and γ-mouse ENaC have been described previously [21].

### Isolation of Collecting Ducts

Patch clamp electrophysiology was used to assess ENaC activity in isolated, split-open rat cortical collecting duct (CCD). Pathogen-free Sprague-Dawley rats of either gender (3 to 4 weeks) were purchased from Charles River Laboratories (Wilmington, MA). This preparation has been described previously [34]–[36]. In brief, freshly isolated kidneys were cut into thin slices (<1 mm). Collecting ducts were mechanically isolated from these slices by micro-dissection using forceps under a stereomicroscope. Isolated cortical collecting ducts were allowed to settle onto 5×5 mm coverglass coated with poly-L-lysine. To gain access to the apical membrane CCD were split open with a sharpened micropipette controlled with a micromanipulator.

### Membrane Labeling Experiments

Membrane labeling experiments followed those described previously [20]. In brief, CHO cells were transfected with Myc-tagged ENaC. 24 hrs post transfection cells were treated with CytD for 10–20 min. Immediately after the treatment cells were washed twice with ice-cold Ca^2+^ and Mg^2+^ containg PBS (pH 8.0) and subsequently incubated with 0.5 mg/ml sulfo-NHS-LC-biotin (Pierce, Rockford, IL) using same buffer for 30 min at 4°C in the dark. Biotinylation was quenched by washing cells with 100 mM glycine containing PBS. Cells were harvested by scraping in 1.0% Nonidet P-40 containing gentle lysis buffer (GLB) supplemented with complete mini protease inhibitor cocktail tablet (Roche, Indianapolis, IN). Brief pulse sonication was used on samples to ensure efficient lysis. After sonication cells were spin cleared for 3 min at 10,000×g and normalized for total protein concentration using DC protein assay (BioRad, Hercules, CA). Pre-equilibrated in GLB streptavidin-agarose beads (Pierce, Rockford, IL) were agitated for 1 hr at 4°C with 300 µg of total protein. Agarose beads were then washed and bound protein was eluted in reducing SDS sample buffer by 10 min boiling. Samples were run on 7.5% polyacrylamide gels in the presence of SDS, transferred to nitrocellulose, and probed with anti-Myc antibody in Tris-buffered saline supplemented with 1% dried milk and 0.1% Tween 20.

### Electrophysiology

For electrophysiological experiments CHO or HEK293 cells were seeded on sterile 4×4 mm cover glasses in 35 mm Petri dishes and transfected using Polyfect reagent (Qiagen, Valencia, CA) as described previously [37]. To define successfully transfected cells 0.5 µg of plasmid containing eGFP was also added to cDNA mix. Single-channel and whole-cell current data were acquired and subsequently analyzed with an Axopatch 200B amplifier (Axon Instruments) interfaced via a Digidata 1440A to a PC running the pClamp 9.2 or 10.2 suite of software (Axon Instruments). Typical bath solution was (in mM): 150 NaCl, 1 CaCl_2_, 2 MgCl_2_, 10 HEPES (pH 7.4). Pipette solutions for cell attached and whole cell configurations were (in mM): 140 LiCl, 2 MgCl_2_ and 10 HEPES (pH 7.4), and 120 CsCl, 5 NaCl, 2 MgCl_2_, 5 EGTA, 2 Mg-ATP, 0.1 GTP, 10 mM HEPES (pH 7.4) respectively. Gap-free single channel current data from gigaohm seals in HEK293, mpkCCD_c14_ and rat CCD cells were acquired and subsequently analyzed and low-pass filtered at 100–200 Hz with an eight-pole Bessel filter (Warner Instruments). *NP_o_*, the product of the number of channels and the open probability (*P_o_*), or *P_o_* itself, were used to measure the channel activity within a patch. Single-channel unitary current (i) was determined from the best-fit Gaussian distribution of amplitude histograms. Channel activity was analyzed as *NP*_o_ = *I*/*i*, where *I* is mean total current in a patch and *i* is unitary current at this voltage. When multiple channel events were observed in a patch, the total number of functional channels (N) in the patch was determined by observing the number of peaks detected on all-point amplitude histograms.

Whole-cell macroscopic current recordings of mENaC expressed in CHO cells were made under voltage-clamp conditions using standard methods [17], [37]. Cells were clamped to a 40 mV holding potential with voltage ramps (500 ms) from 60 mV down to −100 mV used to elicit current. ENaC activity was assessed as the amiloride-sensitive current density at −80 mV. Whole-cell capacitance, on average 6–10 pF, was compensated. Series resistances, on average 2–5 MOhm, were also compensated.

### Statistics

All summarized data are reported as means ± SEM. Data are compared using with either the Student's (two-tailed) *t*-test or a one way ANOVA and P<0.05 is considered significant.
